# Stimuli-Responsive Nanofibers Containing Gold Nanorods for On-Demand Drug Delivery Platforms

**DOI:** 10.3390/pharmaceutics13081319

**Published:** 2021-08-23

**Authors:** Baljinder Singh, Nutan Shukla, Junkee Kim, Kibeom Kim, Myoung-Hwan Park

**Affiliations:** 1 Department of Convergence Science, Sahmyook University, Seoul 01795, Korea; vishalmasih94@gmail.com (B.S.); nutanshukla14@gmail.com (N.S.); junkee0215@naver.com (J.K.); 2 Convergence Research Center, Nanobiomaterials Institute, Sahmyook University, Seoul 01795, Korea; kibumsy@syu.ac.kr; 3 Department of Chemistry and Life Science, Sahmyook University, Seoul 01795, Korea; 4 N to B Co., Ltd., Business Incubator Center, Hwarang-ro, Nowon-gu, Seoul 01795, Korea

**Keywords:** on-demand drug delivery systems, electrospun nanofibers, poly (N-isopropylacrylamide), gold nanorods

## Abstract

On-demand drug delivery systems using nanofibers have attracted significant attention owing to their controllable properties for drug release through external stimuli. Near-infrared (NIR)-responsive nanofibers provide a platform where the drug release profile can be achieved by the on-demand supply of drugs at a desired dose for cancer therapy. Nanomaterials such as gold nanorods (GNRs) exhibit absorbance in the NIR range, and in response to NIR irradiation, they generate heat as a result of a plasmon resonance effect. In this study, we designed poly (N-isopropylacrylamide) (PNIPAM) composite nanofibers containing GNRs. PNIPAM is a heat-reactive polymer that provides a swelling and deswelling property to the nanofibers. Electrospun nanofibers have a large surface-area-to-volume ratio, which is used to effectively deliver large quantities of drugs. In this platform, both hydrophilic and hydrophobic drugs can be introduced and manipulated. On-demand drug delivery systems were obtained through stimuli-responsive nanofibers containing GNRs and PNIPAM. Upon NIR irradiation, the heat generated by the GNRs ensures shrinking of the nanofibers owing to the thermal response of PNIPAM, thereby resulting in a controlled drug release. The versatility of the light-responsive nanofibers as a drug delivery platform was confirmed in cell studies, indicating the advantages of the swelling and deswelling property of the nanofibers and on–off drug release behavior with good biocompatibility. In addition, the system has potential for the combination of chemotherapy with multiple drugs to enhance the effectiveness of complex cancer treatments.

## 1. Introduction

On-demand drug delivery systems (DDSs), which are programmable in a patient-friendly manner, can spatially and temporally control drug delivery at a particular site and the rate of drug release over a specific period of time [1,2,3]. Recently, the development of on-demand DDSs from stimuli-responsive nanomaterials, which provide a controlled and pulsatile release of drugs at certain concentrations in the body, has received significant interest [4,5,6]. Conventional DDSs pose some challenges such as difficulty in controlling the drug release rate, unsuitability of the drugs for other body organs, and the production process of the system [7,8]. Several drugs are not appropriate for oral drug delivery, owing to their limitation of drug degradation under the acidic and alkaline conditions of the stomach and intestine, respectively [9,10]. Intravenous injection for drug delivery can resolve some of the problems that occur in oral drug delivery; however, this system also has various issues such as the drug administration requiring professional skill, specific storage issues, and sterility [11,12]. Furthermore, side-effects on nontargeted sites in the body are another major issue in DDSs [13,14]. Therefore, it is necessary to explore an innovative drug delivery platform that can maintain sustained and controlled drug release during the treatment cycle, achieve a targeted drug delivery, and circumvent the abovementioned challenges [15]. 

As a result, implantable drug delivery has emerged as an adequate platform for local drug delivery in cancer by aiming at the tumor site and effectively removing the damage by migrating from the bloodstream to the target tissue [16]. Hence, stimuli-responsive nanomaterials as nanocarriers enhance the therapeutic potential for the development of a platform for effective drug delivery in cancer therapy [17,18,19]. Smart polymeric nanofibers are promising materials for therapeutic platforms with advanced biomedical applications owing to their high biocompatibility, high drug encapsulation efficacy, ease of surface modification, and controllable characteristics [20,21,22]. The nanofibers with anticancer drugs can provide sustained drug release at the local targeted sites [23,24]. The main advantage of local drug delivery at the targeted site is to prevent undesirable side-effects at nontargeted sites, which also enhances the drug efficacy by delivering high concentrations of the drug at the desired sites [25,26]. Therefore, smart DDSs based on such nanofibers are required to enable on-demand release for effective treatment. Thus, the DDSs can attain an anticipated mode of drug delivery by sparing the healthy cells.

Nanofibers can play an essential role in wound dressing and can be a good drug carrier in skin cancer treatment with promising properties such as curing, controlling the chemical environment around the wound area that can maintain proper moisture, and protecting the wound from further damage [27]. In this case, nanofiber-based drug delivery with nontoxic behavior and skin compatibility can be an optimistic system by providing controlled drug release with an effective concentration to the wound site and skin cancer area [28]. On-demand drug release from nanofibers can be achieved based on the choice of responsive materials and encapsulation methods [29,30]. Smart nanomaterials have been widely developed because of their responsive behavior to external stimuli such as pH, temperature, light, and ultrasound [31,32,33,34]. Among all these external stimuli, light has received considerable attention because of its ease of use and better spatiotemporal control [35]. Ultraviolet (UV)-light-responsive systems have disadvantages such as poor tissue penetration and harmful effects on cells and tissues due to the accumulation of DNA damage, and if the body is unable to repair this damage, it can begin to divide and grow in an uncontrolled manner and lead to cancer [36,37]. In contrast, NIR light exhibits good and deep tissue penetration and is also safe for cells and tissues [38,39]. 

Several nanofibers with inorganic nanoparticles such as Au, Ag, Fe_3_O_4_, Si, and graphene oxide introduced inside or on the surface of the nanofibers have been prepared, which play important roles in DDSs [40,41,42,43]. Among them, gold nanorods (GNRs) have attracted significant attention due to their strong absorption in the NIR range (650–900 nm), which is harmless to the human body [44,45]. GNRs are attractive probes for cancer imaging because of their highly effective absorption in the NIR region, a spectral window that permits photons to penetrate biological tissues with relatively high transmittance. Apart from being attractive probes for cancer imaging, the GNRs are also heat-generating sources owing to their surface plasmon resonance (SPR) effect [46,47,48]. In this study, nanofibers were prepared using poly(N-isopropylacrylamide) (PNIPAM)—a temperature-responsive polymer—because it has potential applications in DDSs [49]. PNIPAM causes a reversible phase transition at a specific temperature defined as the lower critical solution temperature (LCST). At temperatures below the LCST, PNIPAM expands in water through intermolecular hydrogen bonding between the polymer chains of PNIPAM and water molecules. These intermolecular hydrogen bonds are replaced by intramolecular hydrogen bonds between CO and NH groups along the PNIPAM chain at temperatures above the LCST. This results in the aggregation of polymers in water. Most of the polymers are not crosslinked; hence, they are easily soluble in water [50]. Nanofibers from the PNIPAM homopolymer are unstable in water; therefore, cross-linking for copolymerization is required to enhance the stability of the nanofibers in an aqueous solution [51,52,53]. The PNIPAM nanofibers containing GNRs exhibit optical sensitivity, and the heat generated by the GNRs can control the swelling and deswelling property due to the thermal sensitivity of PNIPAM [54]. This method can be used in various treatments that generate local heat through NIR irradiation, which can penetrate body tissues to up to 10 cm without serious damage to surrounding tissues [55]. The photothermal effect becomes strong by introducing the porous structures and GNRs inside the nanofiber, and the thermal/optical response speed can be increased by rapidly increasing the temperature above the LCST of PNIPAM. The crosslinked composite nanofibers can be used as an on–off drug release system by simply irradiating the surface with NIR [56].

**Scheme 1:**
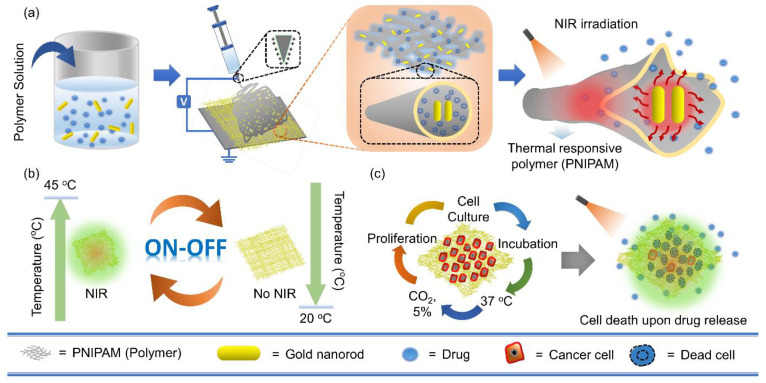
Schematic illustration of stimuli-responsive nanofibers containing GNRs for on-demand drug delivery platform. (**a**) Fabrication of nanofiber through electrospinning technique and drug release upon NIR irradiation. (**b**) On–off cyclic profile of the nanofibers. (**c**) Cell proliferation on the nanofibers and cell death upon drug release.

PNIPAM nanofibers containing GNRs with fast thermal/optical response, high heating rate, and high structural stability were prepared through the electrospinning method [57]. Electrospun nanofibers provide easy surface functionalization in the space between small fibers and have high surface-area-to-volume and porosity mass ratios [58,59]. In electrospinning, when a high voltage is applied to a solution being discharged at a constant speed through a nozzle, it forms a Taylor cone by electrostatic force. Furthermore, the solvent evaporates instantaneously, forming nanofibers with a large surface area in the collector grounded with the polymer [60,61]. Through a simple electrospinning process, therapeutic drugs can be conveniently introduced into nanofibers [62,63]. Until now, studies have been conducted on nanofibers in which drugs are introduced using various substances such as antibiotic, chemotherapeutic, and vitamin substances [64]. However, a DDS using composite materials emerges as a promising platform because nanofibers have a long-time stability due to the presence of drugs and GNRs, which is convenient for the on–off cyclic profile of the drug release (Scheme 1). This method provides efficient loading of low-solubility drugs into the nanofibers and is suitable for the encapsulation and release of hydrophobic and hydrophilic drugs. This ideal system allows drugs to be safely introduced into DDSs and to control drug release to treat cancers or overcome other complex diseases [65,66,67]. The objective of this study was to achieve a platform that addresses problems with conventional release methods, such as insufficient drug release at targeted sites owing to drug waste at nontargeted sites and externally uncontrolled release due to the treatment period that leads to reopening and painful operations [68]. The embedding of GNRs into the matrix of nanofibers elevates them to a new category of biomaterials capable of reacting to stimulation [69]. Using this approach, we developed a method to treat glioblastoma (GBM), also known as a grade IV astrocytoma, a fast-growing and aggressive brain tumor through the externally controlled release of camptothecin (CPT), which can promote a senescence-like phenotype in brain cancer cells [70]. The direct delivery of chemotherapy agents to the brain is a clinically proven method for treating glioblastoma multiforme, but current technologies have significant limitations, including severe local tissue toxicity and a limited diffusional penetration of agents, which limit its application and effectiveness [71]. CPT-loaded nanofibers can be delivered to a stereotactically specified position in the brain, providing the simultaneous control of drug release location, diffusion, and duration in our new method [72]. This CPT analog can improve the efficacy and stability on the tumor site for more effective local anticancer therapies against brain cancers cells [73]. Therefore, we used the U-87 MG cell line in this study. Hence, this study emerges as a novel approach for externally controlled drug release for efficient therapeutic effects in cancer treatment.

## 2. Materials and Methods

### 2.1. Materials

Poly(N-isopropylacrylamide) (MW 300,000 Da, Polysciences, Warrington, PA, USA), octaglycidyl polyhedral oligomeric silsesquioxane (OpePOSS) (Hybrid Plastics Inc., Hattiesburg, MS, USA), 2-ethyl-4-methylimidazole (EMI), gold(III) chloride trihydrate (HAuCl_4_·3H_2_O), cetyltrimethylammonium bromide (CTAB), sodium borohydride (NaBH_4_), silver nitrate (AgNO_3_), ascorbic acid, 11-bromo-1-undecanol (98%), triphenylmethanethiol (97%), methanesulfonyl chloride (MsCl, 98%), trifluoroacetic acid (TFA, ≥99%, liquid), triisopropylsilane (TIS, 98%), and triethylamine (TEA, ≥99%) were purchased from Sigma-Aldrich (St. Louis, MO, USA) and used as received. Other organic solvents required for ligand synthesis were purchased from Sigma-Aldrich (St. Louis, MO, USA). Fluorescein was purchased from JUNSEI (Tokyo, Japan). CPT was purchased from TCI (Tokyo, Japan). The ^1^H-NMR graph was measured with a CDCl_3_ solvent using a JEOL ECX-400 400 MHz (JEOL, Tokyo, Japan) spectrometer. Cells were obtained from the Korean Cell Line Bank (Seoul, Korea). All cell reagents for in vitro studies such as phosphate-buffered saline solution (PBS), Dulbecco’s modified Eagle’s medium (DMEM), fetal bovine serum (FBS), penicillin–streptomycin, and trypsin-ethylenediaminetetraacetic acid (trypsin-EDTA) were all purchased from Sigma-Aldrich (St. Louis, MO, USA). The cell viability was quantified using 3-(4,5-dimethylthiazol-2-yl)-2,5-diphenyltetrazolium bromide (MTT), which was purchased from Sigma-Aldrich (St. Louis, MO, USA). Fluorescence spectra were collected using a Neosys-2000 UV-Vis spectrophotometer (Scinco, Twin Lakers, WI, USA) and a QM-400 spectrophotometer (Horiba Scientific, Piscataway, NJ, USA). A diode laser system with a wavelength of 808 nm and continuous-wave operation mode was used as photo-stimulation, and temperature traces were recorded using a Ti95 infrared camera (Fluke, Washington, WA, USA). The cell viability was measured using a microplate reader (Tecan Trading AG, ZH, Switzerland).

### 2.2. Preparation of Both Organic and Water-Soluble TMA-GNRs

The GNRs used in this study were prepared according to the well-known seed-mediated growth method using CTAB, and the stability of GNRs in organic solvents was ensured through surface modification of the GNRs with HS-C_11_-trimethyl ammonium (TMA), as reported by Jeong et al. [74].

### 2.3. Characterization of GNRs

The morphology of GNRs was investigated by transmission electron microscopy (TEM) (Tokyo, Japan, JEOL JEM-2010). GNRs were dispersed in ionized water (IW). A single drop of GNR solution was applied on a carbon-coated copper grid (200 mesh) and allowed to dry at ambient temperature before imaging with a 50 nm scale bar. The SPR spectra of GNRs were examined using UV–Vis spectroscopy (Scinco, USA) in the wavelength range of 400–1100 nm by adding 2 mL of GNRs solution to a 4 mL quartz cuvette. Zeta potential analysis was determined by adding a maximum of 400 μL of the three different samples to a zeta potential cuvette with a positive and a negative electrode. The equilibration time for each was set at 120 s, with a total of 50 runs. Three separate tests were carried out under these conditions.

### 2.4. Fabrication of Light-Responsive Electrospun Nanofibers

To make 5 mL of polymeric solution for electrospinning, 0.5 g (10%) of PNIPAM, 0.15 g (3%) of OpePOSS, and 0.01 g (0.2%) of EMI were dissolved in 4 mL of DMF:THF 1:1. The mixture was stirred for 4 h at room temperature. Finally, 1 mL of TMA-GNRs DMF:THF 1:1 solution (200 nM) was added to the obtained solution, and the mixture was stirred for an additional hour to form a uniform solution. A certain polymer concentration that produces the nanofibers of the same diameter and suitable uniformity was chosen. If the concentration is low, the diameter of the resulting fibers becomes nonuniform and some bonds or even the fibers may not be formed. Conversely, at higher concentration, the diameter of the fibers is large; hence, the rate of penetration of water is slow, which affects the response speed of the composite film. The resulting homogeneous polymer solution was injected into a 10 mL plastic syringe. During electrospinning, a direct-current voltage of 13.5 kV was applied to the needle, and the polymer solution was supplied at a flow rate of 0.05 mL/min at room temperature. Furthermore, the distance between the needle and the collection plate was 12 cm. The electrospinning process was performed at 26.4 °C and 45–50% relative humidity (RH) measured by a thermo hygrometer. The prepared nanofibers were placed in a vacuum oven at 160 °C for 4 h to crosslink the PNIPAM nanofibers.

### 2.5. Characterization of Nanofibers

The morphologies of nanofiber scaffolds were analyzed using a scanning electron microscope (SEM) (Tokyo, Japan, JEOL JSM-6510) and confocal laser scanning microscopy (CLSM) (Solms, Germany, Leica TCS SP8). The nanofibers containing GNRs and fluorescein were prepared and cut into circles of 1 cm diameter using a biopsy punch. The nanofiber samples were coated with 250 Å of gold via a Denton Desk V Sputter Coater. The SEM images were obtained at an accelerated voltage of 20 kV and 20 μm scale bar. Fiber diameter distribution histograms were quantified using the SEM micrographs. For each sample, 10 random field images were taken, and 10 fibers were measured in each image. The samples from the same nanofibers with a diameter of 1 cm were taken for CLSM. In this analysis, two samples were prepared that were the original dry nanofiber and water-treated nanofiber. The CLSM images were obtained under 63× magnification. All these characterizations were observed at room temperature.

### 2.6. On-Demand Drug Release

To study the behavior of NIR-responsive drug release at different irradiation times, nanofibers containing GNRs and fluorescein (model drug) were prepared. The nanofibers were cut into circles of 1 cm diameter using a biopsy punch and added in 1.5 mL of IW. To check the fluorescence intensity of drug release, the sample tube, which was placed 10 cm from the center of a laser probe, was irradiated directly on the nanofiber sample by a diode laser (808 nm) at a laser power of 1.6 W/cm^2^ up to 60 min at 10 min intervals. The drug release was confirmed by fluorescence spectroscopy after every 10 min of the same sample tube. The pulsatile drug release was performed in a cyclic on–off manner by 10 min of no NIR light irradiation and 2 min of NIR light irradiation at a laser power of 1.6 W/cm^2^ up to 60 min, and the solution was quantified via a QM-400 spectrophotometer after 10 min of no irradiation and 2 min of irradiation. Furthermore, the cumulative drug release with 0.6 W/cm^2^, 1.1 W/cm^2^, and 1.6 W/cm^2^ laser powers was performed. In this experiment, four different samples were used, and the laser power irradiated up to 60 min at 5 min intervals. For all these drug release experiments, all the samples containing the same amount of drug (2 μg) were used and the experiment was performed at room temperature.

### 2.7. Biocompatibility and Toxicity

Cell studies were performed to evaluate the biocompatibility and toxicity of the nanofibers containing GNRs and fluorescein using the U-87 MG (brain cancer cells) cell line. The nanofibers were cut into circles of 1 cm diameter using a biopsy punch and attached to the bottom of a 24-well plate. Prior to cell seeding, all wells containing nanofibers were preconditioned overnight in DMEM containing 10% FBS and 1% penicillin/streptomycin at 37 °C in a 5% CO_2_ incubator. Thereafter, the media were refreshed and the cells with a density of 3 × 10^3^ cells/well were cultured on the nanofibers under the same conditions mentioned above. After 24 h, the supernatant was discarded, and the cells were incubated with trypsin-EDTA for 5 min to detach from the surface of the nanofibers for cell counting.

### 2.8. MTT Assay for Cell Viability

PNIPAM nanofibers containing GNRs and CPT were prepared. In addition, 1 cm-diameter circles of the nanofiber samples were used, each of which contained 30 μg of payload. Further, 500 cells/well were incubated in a 96-well plate for 24 h in DMEM containing 10% FBS and 1% penicillin/streptomycin at 37 °C in a 5% CO_2_ incubator before experiments. The samples were irradiated with NIR light at different laser powers for 2, 10, and 20 min to release the drug from the nanofibers to the cells in the 96-well plate. After 2–4 h or incubation, the MTT assay was conducted using a microplate reader at 570 nm absorption. In this analysis, three control groups such as only cells without nanofibers (cells w/o NFs), nanofibers containing GNRs without CPT (NFs+GNRs), and nanofibers containing GNRs with CPT (NFs+GNRs+CPT) were prepared. The test group analysis was performed based on different laser powers. The laser powers used in this experiment were 0.6, 1.1, and 1.6 W/cm^2^.

### 2.9. Data and Statistical Analysis

All statistical analyses were performed using ANOVA analysis with a test level set at *p* ≤ 0.05, which was considered to be a statistically significant difference. The results of all numerical variables were examined by the statistical mean, standard deviation, and graphical analysis using GraphPad Prism 9.2.0 software (GraphPad Software, San diego, Inc., CA, USA). All cell viability tests were performed with three independent samples from each group for all the different assays.

## 3. Results and Discussion

**Figure 1:**
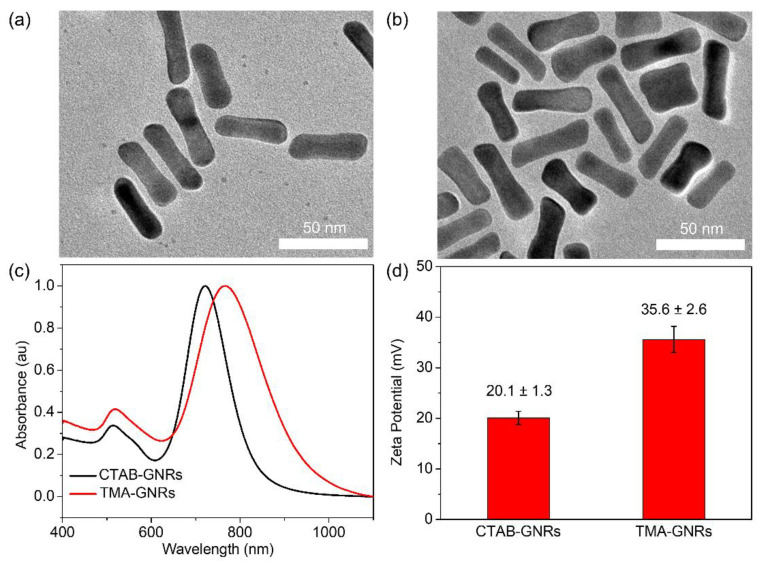
TEM images of (**a**) CTAB-GNRs and (**b**) TMA-GNRs; (**c**) UV-Vis spectra of CTAB-GNRs and TMA-GNRs; and (**d**) zeta potential of CTAB-GNRs and TMA-GNRs.

The plasmon-based photothermal effect of GNRs has become an excellent source of controlled drug release with potential applications and outstanding properties in DDSs. As mentioned earlier, GNRs have strong absorption in the NIR wavelength range; hence, in this study, we irradiated GNRs with NIR light to generate heat to shrink the nanofibers, which results in drug release. The GNRs used in this study were prepared using CTAB, which is a toxic surfactant and unstable in organic solvents. Hence, surface modification was performed on the GNRs to ensure good stability in organic solvents. For this purpose, CTAB attached on the surface of the GNRs was exchanged with TMA through the ligand exchange process. The obtained TMA-GNRs exhibited good stability in DMF:THF 1:1, which we used as the electrospinning solution. CTAB-GNRs and TMA–GNRs were characterized through TEM, UV-Vis spectroscopy, and the zeta potential (Figure 1). The TEM images (Figure 1a,b) show that the GNRs retained their rod shape even after exchanging the surface functionality from CTAB to TMA. The UV-Vis spectrum (Figure 1c) of the CTAB–GNRs solution was confirmed to have specific peaks at 513 nm and 722 nm. Furthermore, in the UV-vis spectrum of TMA–GNRs, the peaks corresponding to TMA–GNRs appeared at 519 nm and 765 nm. Due to the change in the dielectric constant of the environment of each GNR and the sensitivity to the different organic solvents used for the spectrum measurement, the absorption spectra of TMA-GNRs showed pronounced shifts in both the transverse and longitudinal surface plasmon resonance (LSPR) bands after ligand exchange. The zeta potential (Figure 1d) value of CTAB-GNRs was measured at 20.1 ± 1.3 mV, whereas that of TMA-GNRs increased to 35.6 ± 2.6 mV owing to the high charge density after surface modification, which indicates that TMA successfully replaced CTAB from the surface of the GNRs.

**Figure 2:**
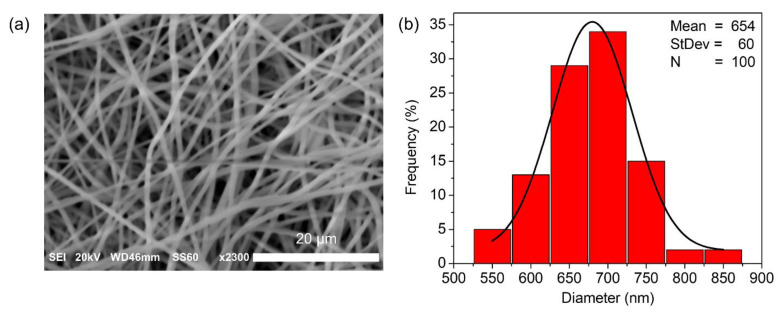
(**a**) SEM images and (**b**) diameter distribution histogram of the nanofibers.

**Figure 3:**
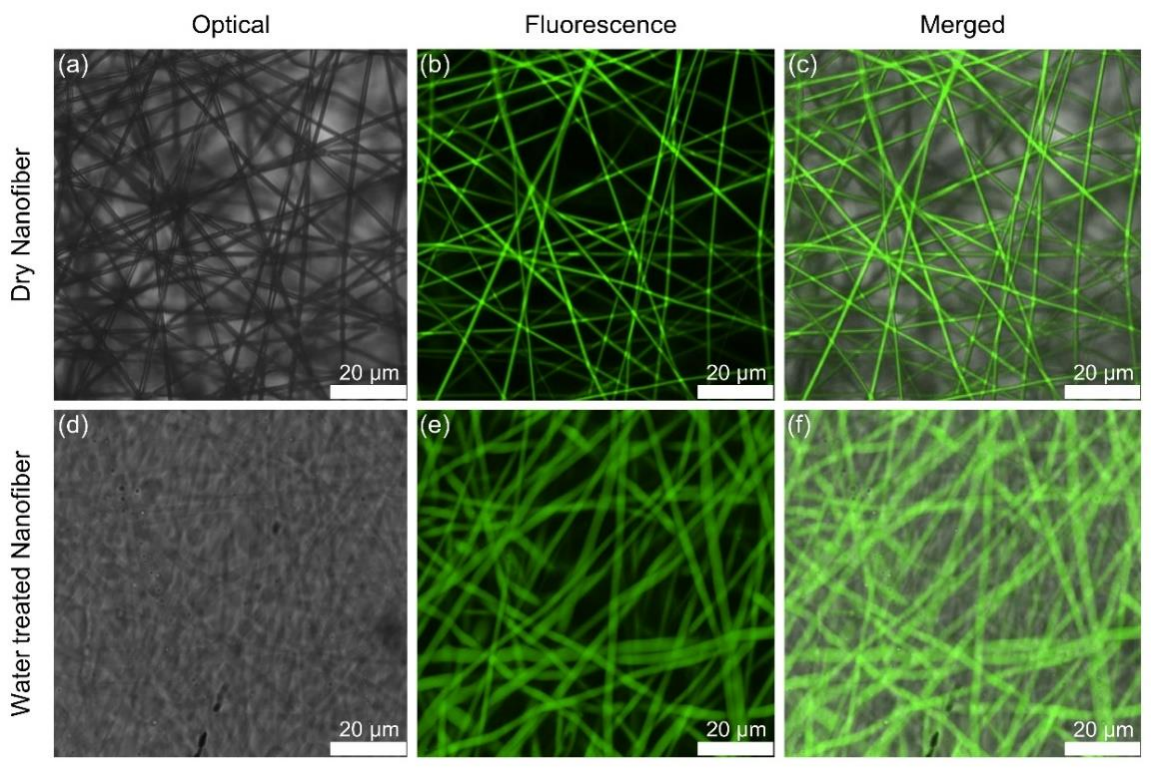
CLSM images of the nanofiber containing fluorescein. (**a**–**c**) Original dry nanofibers. (**d**–**f**) Water-treated nanofibers.

In general, the morphology of nanofibers does not depend only on the electrospinning solution, but also on the parameters of the electrospinning process such as flow rate, high voltage, and distance between the nozzle and the collector. All the parameters used in this method were selected based on the requirements of this study. As shown in Figure 2a, SEM images of the nanofibers obtained the morphology after electrospinning and had an average diameter of 600–700 nm [54]. The nanofibers had a 34% frequency of 700 nm and a 29% frequency of 650 nm, according to the diameter distribution histogram in Figure 2b. As shown in Figure 3, the fluorescein-loaded nanofibers were visualized using CLSM. The CLSM images in Figure 3a show optical images of the dry nanofibers, and a strong green fluorescence matrix is observed in Figure 3b,c, thereby indicating that fluorescein was successfully loaded onto the matrix of the nanofibers. The CLSM of the fluorescein-loaded nanofibers was measured in two different states, that is, original dry nanofibers and water-treated nanofibers. In Figure 3a–c, the dry nanofibers exhibited uniform nanofibrous structures with an average diameter of 600–700 nm. In addition, Figure 3d–f show that the nanofibers were swollen, and their average diameter increased to 2 µm after immersing in water.

**Figure 4:**
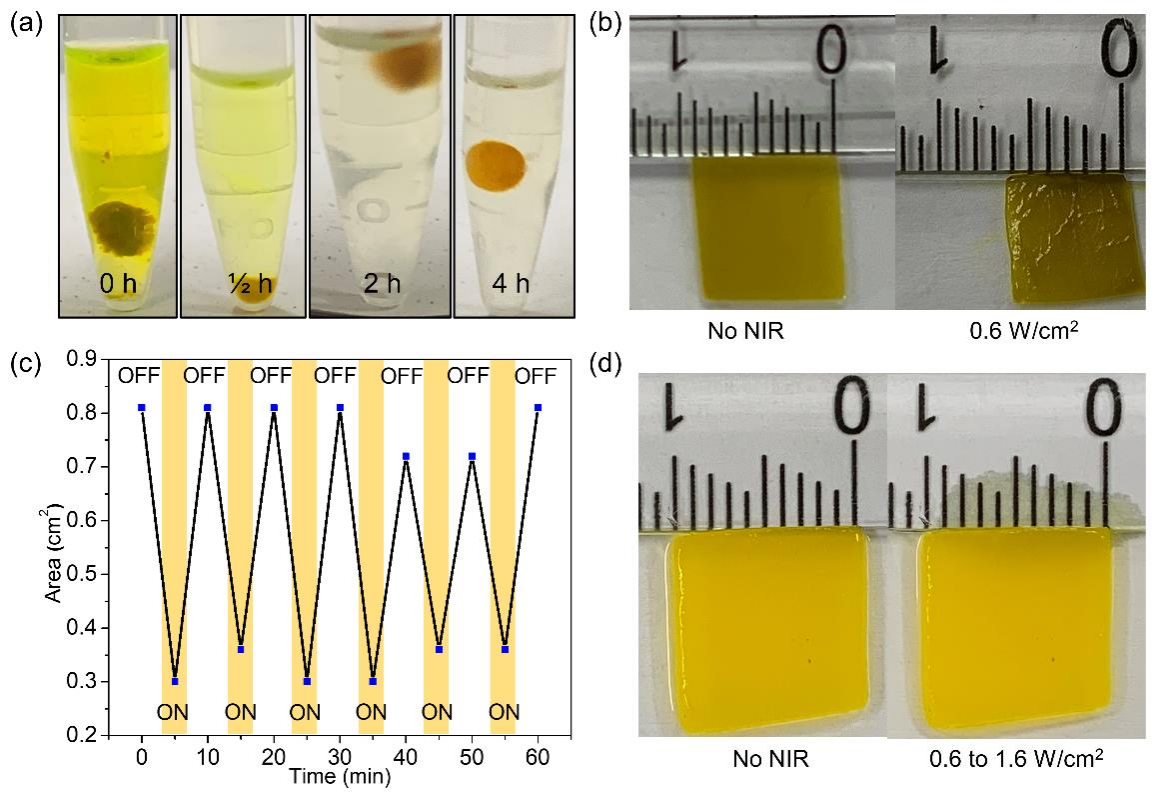
(**a**) Heat treatment of nanofiber under 160 °C. (**b**) Digital images of the area changes of the whole nanofiber containing GNRs upon irradiation of NIR light. (**c**) Change in area of the whole nanofiber containing GNRs as a function of cycles of temperature alternation upon the NIR irradiation. (**d**) Digital images of the area of the whole nanofiber without GNRs in the presence and absence of NIR light irradiation.

The crosslinking of PNIPAM and OpePOSS through heat treatment at 160 °C is a promising method for the high and long-time stability of nanofibers in an aqueous solution. Heat-treatment at 160 °C for 0.5, 2 h, 4 h, and 0 h with four different samples confirmed the cross-linking of prepared nanofibers containing GNRs and fluorescein for on-demand drug release in Figure 4a. The time of 0 h indicates that no heat treatment was provided. As shown in Figure 4a, the nanofibers with 0 h, 0.5 h, and 2 h of heat treatment had low stability with fluorescein leaking in water. However, after 4 h of heat treatment, the high stability of the nanofibers was observed without any fluorescein leakage. Meanwhile, the stimuli-responsive behavior of the nanofibers containing GNRs was investigated using NIR light (Figure 4b). The NIR laser power of 0.6 W/cm^2^ was considered as a minimal power. As shown in Figure 4c, the original nanofibers had a surface area of 0.81 cm^2^; however, when the nanofibers were exposed to the NIR light, they immediately shrank, and their surface area decreased to 0.3 cm^2^. Moreover, Figure 4c demonstrates that when the NIR light was off, the nanofibers returned to the original surface area. These quick and reversible area changes upon the irradiation of the NIR light in a cyclic on–off manner were observed without any significant defects. On the other hand, the nanofibers without GNRs did not respond to the NIR light (Figure 4d), even after increasing the laser power from 0.6 W/cm^2^ to 1.6 W/cm^2^, indicating that the system had a strong photothermal effect owing to the presence of the GNRs. These results also confirmed the presence of GNRs in the matrix of the nanofibers.

**Figure 5:**
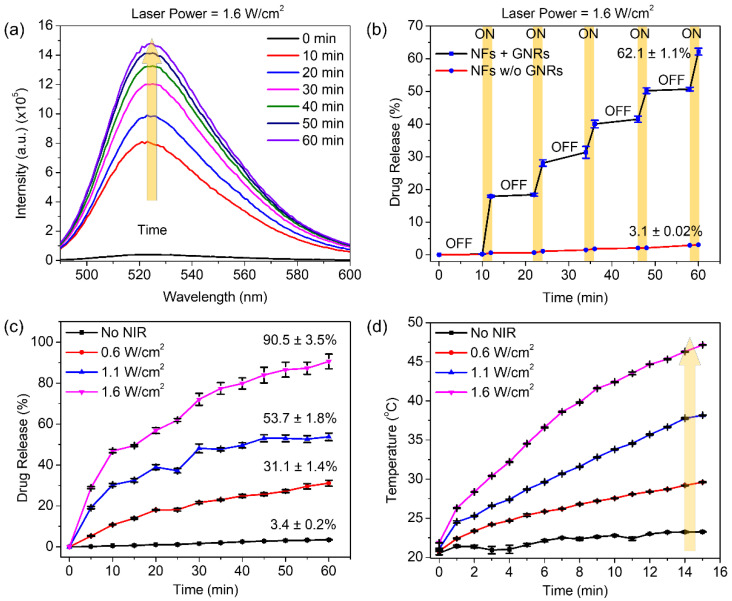
(**a**) Fluorescence intensity of drug release. (**b**) Pulsatile drug release from the nanofibers through the cyclic on–off of NIR light irradiation at different time intervals. (**c**) Cumulative drug release from the matrix of the nanofibers with different laser powers. (**d**) Rate of change in temperature for different laser powers.

The characteristics of the controlled drug release, as a photothermal response to the irradiated NIR light, from the nanofibers containing GNRs and fluorescein were confirmed with a fluorescence spectrophotometer (Figure 5). The NIR-triggered drug release from the nanofibers was monitored (Figure 5a). The nanofiber samples of 1 cm diameter with 2 μg of fluorescein (a model drug) were immersed in 1.5 mL of water at room temperature, and the NIR laser with a power of 1.6 W/cm^2^ directly irradiated the nanofibers. The drug release was observed every 10 min. The drug release intensity increased after every 10 min upon NIR irradiation. The thermal response to the NIR light by the GNRs was confirmed. When the nanofibers were not irradiated with the NIR light, there was a slight emission of the drug; hence, the slope of the fluorescence graph was low. However, when the nanofibers were irradiated with the NIR light, the drug was released, and the slope of the fluorescence graph increased rapidly. The on–off drug release profile was further confirmed (Figure 5b). The on–off mechanism was performed in the sequence of 10 min of no NIR light irradiation and 2 min of NIR light irradiation (of power 1.6 W/cm^2^). This cyclic process showed that 62.1 ± 1.1% of drug was released until 60 min. In each step, the nanofibers shrunk owing to the increase in temperature upon NIR light irradiation and swelled when we turned off the NIR light. This process ensured the swelling and deswelling property of the nanofibers [75]. Different laser powers were used to investigate the drug release behavior from the matrix of the nanofibers. The different laser powers exhibited different release rates and amounts of drugs. The laser powers 0.6, 1.1, and 1.6 W/cm^2^ resulted in drug releases of 31.1 ± 1.4%, 53.7 ± 1.8%, and 90.5 ± 3.5%, respectively, as shown in Figure 5c. In the absence of light, a small amount of drug release was observed from the nanofibers, indicating that the GNRs played an essential role as a heat-generating source. These findings appeared to be appropriate for biomedical practice with a drug release of more than 90% within 60 min [76].

**Figure 6:**
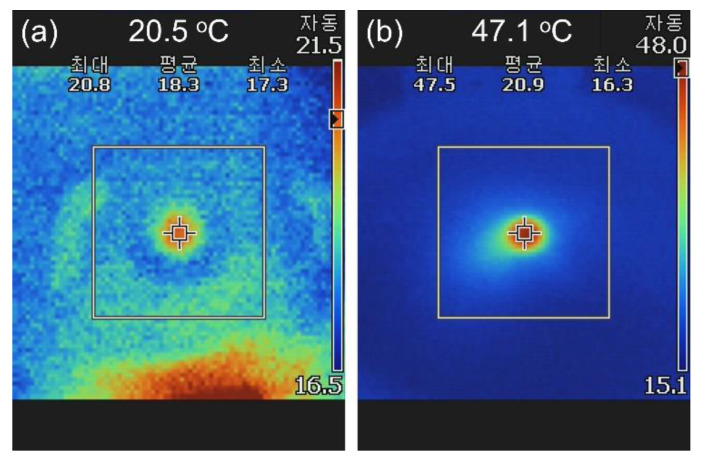
Infrared camera images of the nanofibers (**a**) without GNRs and (**b**) with GNRs.

In addition, the NIR thermal response characteristics of the nanofibers were further confirmed. It was observed that the increase in water temperature depended on the power of the NIR laser light source, and the water temperature increased above 45 °C when irradiated with the NIR laser with a power of 1.6 W/cm^2^. As shown in Figure 5d, upon 15 min of NIR laser irradiation of powers 0.6, 1.1, and 1.6 W/cm^2^, the water temperature increased to the average values of 29.6 ± 0.05, 38.1 ± 0.03, and 47.1 ± 0.03 °C, respectively. These results confirm the capability of hyperthermia therapy for cancer treatment. When the nanofibers were placed in water at room temperature and the NIR laser irradiated the nanofibers, the increase in the water temperature was recorded using an infrared camera (Figure 6). Furthermore, the nanofibers did not increase the water temperature in the absence of GNRs or light.

**Figure 7:**
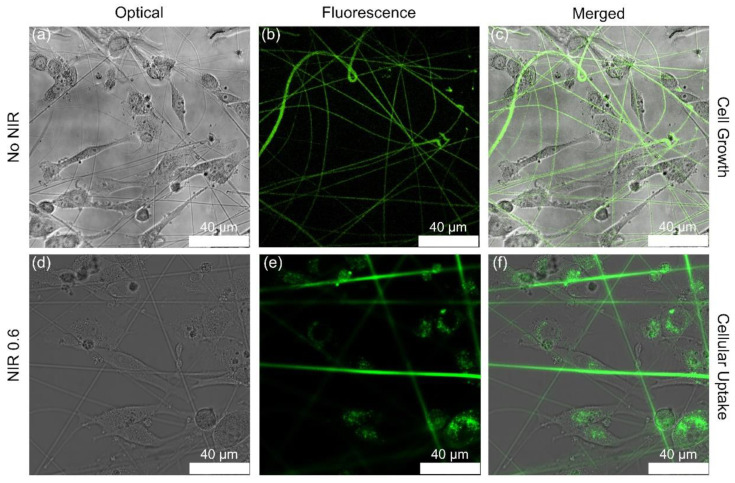
CLSM images of cell proliferation and cellular uptake on the surface of the nanofibers. (**a**–**c**) Cells on the surface of the nanofibers (no NIR irradiation). (**d**–**f**) Cellular uptake after 6 h of incubation and 5 min of NIR irradiation at 0.6 W/cm^2^.

The biocompatibility and toxicity of the fluorescein-loaded nanofibers were evaluated through cell studies. The nanofiber samples were preconditioned before adding the cells. The U-87MG cells with a density of 3 × 10^3^ cells/well were cultured in a 24-well plate without the nanofibers as a control and on the surface of the nanofibers in a DMEM solution at 37 °C in a 5% CO_2_ incubator. The cell growth and morphology were monitored for 24 and 48 h. The cells maintained a proper morphology even on the surface of the nanofibers, similar to that observed in the controls. After 48 h, the supernatant was discarded, and the cells were incubated with trypsin-EDTA for 5 min to detach the cells. The number of adhered cells increased by 508 × 10^3^ cells/well in the control and 431 × 10^3^ cells/well on the nanofibers, demonstrating the capability of easy cell growth on the surface of the nanofibers. Furthermore, according to the results, nanofibers were found noncytotoxic in the absence of NIR light owing to successful cell proliferation (Figure 7a–c).

The cellular uptake was evaluated through cell culturing with a density of 3 × 10^3^ cells/well on the nanofibers, and the cells were incubated for 24 h under the same conditions mentioned above. The cellular uptake of released fluorescein was measured using CLSM in the absence and presence of NIR light. First, the nanofibers were not exposed to NIR light, which resulted in no release of fluorescein. As shown in Figure 7a–c, fluorescence intensity was not observed in the cells after 6 h of incubation. Then, the NIR light (0.6 W/cm^2^) irradiated the nanofibers for 5 min, which provided the fluorescence intensity inside the cells, indicating the release of fluorescein upon NIR light irradiation (Figure 7d–f) [77,78].

**Figure 8:**
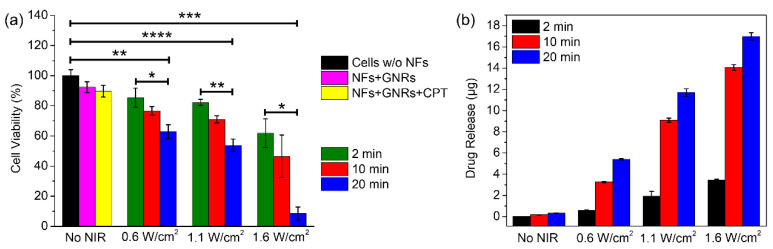
(**a**) Cell viability of U87 cells owing to CPT release from the nanofibers upon NIR irradiation at different time intervals. (**b**) Amount of drug release from the nanofibers upon NIR irradiation at different time intervals. * *p* ≤ 0.05, ** *p* ≤ 0.01, *** *p* ≤ 0.001, **** *p* ≤ 0.0001.

**Figure 9:**
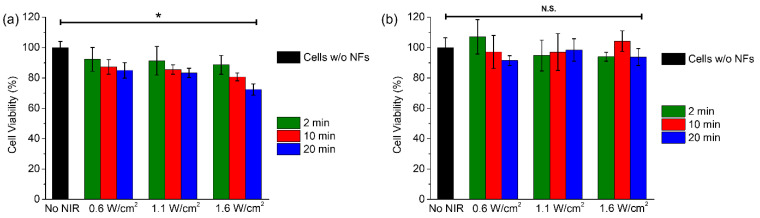
(**a**) Hyperthermia evaluation using the nanofibers containing GNRs with U87 cells. (**b**) NIR light toxicity for the U87 cells in the absence of the nanofibers. * *p* ≤ 0.05. N.S. indicates that the difference was not significant.

MTT assay was performed to evaluate the therapeutic potential of our platform through cell viability with U87 cells, and CPT was chosen as an anti-cancer drug. Cells were incubated for 24 h at 37 °C in a 5% CO_2_ incubator before experiments. In this analysis, three control groups were prepared as: only cells without nanofibers (cells w/o NFs), nanofibers containing GNRs without CPT (NFs+GNRs), and nanofibers containing GNRs with CPT (NFs+GNRs+CPT). Figure 8a shows that there was no substantial decrease in cell viability (approximately 7.6 ± 3.6%) in the case of NFs+GNRs, whereas NFs+GNRs+CPT showed a 10.2 ± 3.9% decrease in cell viability. The nanofibers containing both GNRs and CPT showed maximum cell death upon irradiation of different laser powers after drug release. According to the results, upon NIR light irradiation at 0.6 W/cm^2^ for 2, 10, and 20 min, the cell viabilities were 85.3 ± 6.4%, 76.6 ± 2.7%, and 62.8 ± 4.7%, respectively. Upon the NIR irradiation at 1.1 W/cm^2^ for 2, 10, and 20 min, the cell viabilities were 82.2 ± 2.03%, 70.9 ± 2.4%, and 53.7 ± 4.1%, respectively. Furthermore, upon the NIR light irradiation at 1.6 W/cm^2^ for 2, 10, and 20 min, the cell viabilities were 61.8 ± 9.5%, 46.4 ± 14.1%, and 8.5 ± 4.3%, respectively. The highest cell death was achieved upon increasing the irradiation time and laser power. In Figure 8b, the amount of released CPT has been reported. As shown in Figure 8b, without NIR light irradiation for 2, 10, and 20 min, no significant amount of drug was released. Upon NIR light irradiation at 0.6 W/cm^2^ for 2, 10, and 20 min, 0.6 ± 0.01, 3.2 ± 0.04, and 5.4 ± 0.07 μg of drug were released, respectively. At irradiation with 1.1 W/cm^2^ of laser power for 2, 10, and 20 min, the released amounts were 1.2 ± 0.4, 9.0 ± 0.2, and 11.6 ± 0.36 μg, respectively. Moreover, upon the NIR light irradiation at 1.6 W/cm^2^ for 2, 10, and 20 min, the drug release amounts were 3.4 ± 0.06, 14.0 ± 0.2, and 16.9 ± 0.35 μg, respectively. The hyperthermia effect was obtained due to the presence of GNRs in nanofibers. Cell viability decreased as the NIR laser power increased, as seen in Figure 9a. As the GNRs were present in the matrix of the nanofibers, the hyperthermia effect weakened owing to the poor exposure of the GNRs to the cancer cells. As a result of the hyperthermia treatment, the number of dead cells increased as the temperature increased above 40 °C. The most severe hyperthermia effects were observed in the case of NIR irradiation at 1.6 W/cm^2^, that is, as the temperature increased in the range from 41 °C to 45 °C, the cell viability decreased to 88.6 ± 6.2%, 80.7 ± 2.6%, and 72.3 ± 0.05% after 2, 10, and 20 min of NIR light exposure, respectively [79,80]. The toxicity of the NIR light was also investigated (Figure 9b). There was no significant reduction in the cell viability when the cells were exposed to the NIR light at 0.6, 1.1, and 1.6 W/cm^2^, thereby indicating that the NIR light was less toxic to the cells. Based on these findings, we conclude that our method appears to be promising for on-demand drug release and therapeutic efficacy in cancer treatment.

## 4. Conclusions

Herein, we developed PNIPAM nanofibers containing GNRs and drugs that can control the drug release through NIR light irradiation. As CTAB–GNRs are stable only in water and are not dispersed in organic solvents, TMA–GNRs were prepared through an exchange reaction with TMA ligands that are well-dispersed in organic solvents. To prevent the PNIPAM nanofibers from dissolving in water below the LCST, stable PNIPAM nanofibers were prepared through a crosslinking reaction with OpePOSS. The PNIPAM nanofibers containing GNRs and drugs obtained through electrospinning have high thermal/optical responsiveness. In this study, the on-demand drug release was achieved through our versatile nanofiber platform. The results showed that the fabricated nanofibers are structurally stable and have a very large surface-area-to-volume ratio for effective delivery of drugs. A strong photothermal effect was observed by introducing the GNRs in the nanofibers. The heat generated by the GNRs upon NIR light irradiation could control the swelling and deswelling property of the nanofibers owing to the thermal sensitivity of PNIPAM, which results in drug release. This optimal method allows both hydrophilic and hydrophobic drugs to be safely introduced into DDSs and control the drug release to treat cancers and other complex diseases. Through cell studies, good biocompatibility of the nanofibers was confirmed. Furthermore, our method may contribute to the application of the sequential release of multiple drugs, which is the scope of our future studies.

## Data Availability

The data presented in this study are available on request from the corresponding author. The data are not publicly available due to institutional policies.
